# “Do good and talk about it”: informal representation and migrant-led civil society organizations’ mediation between low-wage labor migrants and state institutions in the GCC countries

**DOI:** 10.1186/s40878-024-00417-9

**Published:** 2024-12-18

**Authors:** Mira Burmeister-Rudolph

**Affiliations:** Department of Political Science, Nieuwe Achtergracht 166, Amsterdam, 1018 WV The Netherlands

**Keywords:** Civil society organizations, Citizenship, Labor migration, Precarity, India, Gulf countries, Mediation

## Abstract

Low-wage labor migrants experience major human and working rights abuses in the Gulf Cooperation Council countries despite national labor laws and signatures to various human rights conventions. On paper, India has established an institutional framework of transnational social protection for its officially estimated 5.5 million low-wage workers migrating to the Gulf Cooperation Council countries, e.g., financial emergency support, repatriation services, and walk-in centers. However, migrants in low-wage employment often cannot access substantive social rights in practice. Indian upper/middle-class migrant civil society groups mediate their access to Indian embassies’ services and the destination countries’ state institutions. The realization of social rights via informal, third-party representation stems from a representational disjuncture between low-wage labor migrants and the Indian state, which is rooted in their historical socioeconomic marginalization, limitations of the formal political system, and the constitutive role of informality in shaping and structuring citizen-state interactions in India. Through the lens of Piper and von Lieres’ (2015) concept of mediated citizenship and based on data from semi-structured interviews and participant observation of migrant support networks in the Gulf countries, this article examines why mediation takes place and how volunteers speak and take action for marginalized migrants in low-wage employment and consequences of mediation. It argues that migrant volunteer organizations and individuals are powerful stakeholders in the migration governance in the GCC region, as they possess leverage over who has (better) access to state institutions and the provision of social and human rights. Their status as intermediaries underlines the disaggregation of Indian citizenship along class and caste lines, which can be (mis)-used by mediators to pursue their interests, resulting in ambiguous effects. The article contributes to perspectives on migration governance in the GCC region, transnational social policies, and migrant volunteering.

## Introduction

The Gulf Cooperation Council (GCC) countries have been a key destination for Indian labor migrants since the 1970s and host an estimated 7.9 million out of 15.8 million Indian citizens residing abroad (Ministry of External Affairs, 2024). About 70% of Indian nationals in the GCC countries work in low-wage employment (Khadria, 2006), such as in construction, agriculture, or as domestic workers (Abraham 2012). Similar to low-wage employed migrants worldwide (see, e.g., Neuhauser et al., 2023; Nuti, 2018), the combination of low-wage work and migrant status leaves these workers particularly susceptible to exploitation and the violation of their labor and social rights. These include, e.g. unpaid or underpaid wages, working excessive hours and under dangerous conditions, living in overcrowded accommodations, and the confiscation of their identity documents (Fernandez, 2021). Despite GCC countries’ national labor laws and their signatures to various human rights conventions (Barbar 2020), these countries do not provide (low-wage) labor migrants any social protection.

On paper, the Indian federal state has established an institutional framework of transnational social protection for its international low-wage labor migrants. Among others, mandatory insurance covers costs for medical treatments, accidental death or permanent disability of the worker, or legal representation in work-related disputes; the Indian Ministry of External Affairs established the Community Welfare Fund, which provides financial support for migrants on a case-to-case basis, e.g., for paying fees when migrants overstayed their visa so they can leave the host country (for an overview see Table 1). Embassies and consulates also offer assistance and protection for their citizens in cases of arrest, detention, or loss of passports (see Lafleur & Vintila, 2020), the latter mostly related to the frequent practice of employers to confiscate passports. However, in practice, migrant workers often cannot claim these social provisions for several reasons. For example, they cannot visit embassies due to their inability to leave their accommodation because of long working hours, a lack of public transport, or the fear of being deported when reporting to official institutions, such as an embassy. As a result, low-wage labor migrants often depend on (Indian) upper/middle-class migrant-run civil society organizations^1^’ (CSOs) and individual volunteers’ mediation to access (Indian) embassies, their legal and administrative services, and destination countries’ institutions, such as labor regulations authorities. These volunteers are in charge of distributing Indian government funds and deciding who is eligible for financial support. These and other CSOs – many of which run independently from the Indian embassy – arrange return flights for severely ill migrants and visit mortuaries to identify migrants’ mortal remains; provide legal and psychological support, food, and medical check-ups.

**Table 1:** Overview of Social Protection Schemes by the Ministry of External Affairs, Government of India

	Social Assistance	Labor Market Programs	Social Insurance
**Pre-departure**	**Overseas Workers Resource Centre (OWRC)** (2004) in Delhi, Chennai, Lucknow, Kochi and Hyderabad: • Grievance registration • Tracking of grievance • Assistance for online Migration Clearance (e-Migrate) • Toll-free 24 × 7 helpline support in various Indian languages	**One-day pre-departure training** (2018)	
**Destination Country**	**Indian Community Welfare Fund (Emergency Fund)** (2009) • Assisting in distressed situations • Support for Community Welfare activities • Improvement in Consular services ***Pravasi Bharatiya Sahayata Kendra (PBSK) (2010)*** in Dubai and Sharjah (UAE), Riyadh and Jeddah (Kingdom of Saudi Arabia); Kuala Lumpur (Malaysia) • 24/7 helpline • walk-in counter in Dubai and Sharjah, legal, psychological and financial counseling • awareness campaigns in workers’ accommodation sites **Online Grievances complaint form (MADAD)** against Indian embassy services (2015)		**Compulsory Insurance Scheme** / Pravasi Bharatiya Bima Yojana (PBBY)) (2003): • Cover sum accidental death/permanent disability • One-way ticket for rehabilitation in case of death or disability • Medical treatment for migrant and their families in case of illness • Maternity expenses • Legal expenses related to employment abroad
**Return**			**Voluntary Pension scheme (discontinued in 2017)**: **Mahatma Gandhi Pravasi Suraksha Yojana (MGPSY) (2012)**

I use Piper and von Lieres’ (2015) concept of mediated citizenship to conceptualize the specific mode of relationship between international Indian low-wage migrants, migrants volunteering in CSOs, and the Indian state and to read the complex constellations of migrant-run CSOs’ rights claiming and negotiating on behalf of low-wage labor migrants. Mediation describes third-party actors’ informal representational practices for poor and marginalized citizens to the state and vice versa through pragmatic politics of bargaining and negotiating rather than confrontational contentious politics (Piper & von Lieres, 2015). Mediation occurs when “more direct means of state access through politicians and civil servants is difficult, and/or when democratic mobilization through social movements is absent.” (Piper & von Lieres, 2015, p. 698).

The India-GCC migration corridor is a particularly well-suited case to study practices of mediated citizenship, as Indian migrants often find themselves in low-wage employment and marginalized positions throughout the migration cycle and experience difficulties in directly accessing state institutions and provisions in both the destination countries and the migrant origin contexts. Access to social, political, and economic citizenship rights for marginalized groups is fragmented in India (Jayal, 2009). Consequently, intermediaries, often CSOs, are central in bridging the gap between socio-economically marginalized citizens and the state (Chandhoke, 2012). Furthermore, the Indian federal states’ migration governance of low-wage labor is characterized by exclusions as migrants are often unable to access substantive citizenship rights, such as taking part in elections, and are mostly ignored at the symbolic level (Burmeister-Rudolph, 2023). I demonstrate in this article that CSOs take on similar mediating functions outside the Indian polity to enable low-wage labor migrants’ access to Indian state institutions, here mostly embassies and foreign missions, relevant institutions of the destination countries, and rights, often in the form of social and labor rights, such as health services, adequate accommodations, and paid wages. I thereby expand the concept of mediated citizenship by operationalizing the framework in a transnational setting and showcasing that in an international context, mediation goes beyond claiming citizenry rights but also involves labor rights and bridging representational gaps between non-citizens and the destination states.

Drawing on data from participant observation of migrant support networks in Bahrain and Dubai and semi-structured interviews conducted in early 2020 and 2022 with members of such CSOs and individual persons in Bahrain, Dubai, Qatar, Kuwait, Oman, and India, this article examines why mediation takes place and how volunteers speak and take action for migrants in low-wage employment and the consequences of mediation. It shows that mediation as an informal representational practice has ambiguous effects: while it helps migrants in precarious positions to access labor and social rights, it reproduces social class- and caste-based hierarchies and exclusions. In line with scholarship questioning humanitarianism as an impartial practice (e.g., Fassin, 2012; Ticktin, 2011) but which views it as entangled with the governance of migration, I argue that migrant volunteer organizations and individuals are influential stakeholders in the migration governance in the GCC region, as they possess leverage over who has (better) access to state institutions and the provision of social and human rights.

This study contributes to the broader literature on the politics of migration governance in multiple ways: Based on empirically original material, it sheds light on how migration governance in the GCC region works on the meso-level by highlighting the role of immigrant organizations as civil society stakeholders and thereby complements existing research on the GCC countries, which has been predominantly focusing on states’ regulation of labor migration (Barbar 2020; Thiollet, 2019) and immigrants’ strategies in dealing with the kafala system (e.g., Akıncı, 2022; Vora, 2013). Furthermore, this research questions conceptualizations of volunteering for migrants as either representing non-contentious care relationships (see Herrmann, 2020) or contentious, transformative acts of migrant solidarity (Della Porta & Steinhilper, 2021) because these derive mainly from empirical cases within Europe where volunteers often hold European nationality, and their actions take place within a democratic legal framework. Notably, GCC countries prohibit political associations, and freedom of opinion and expression is limited. Most of the volunteers active in migrant CSOs in the GCC region are immigrants whose residency status is temporary and tied to their employment. The illiberal political context limits space for advocacy as migrant volunteers can face potential deportation when overstepping red lines, such as open protest through marches (see Illias 2020; Thiollet, 2019). Finally, this article adds to scholarship on transnational social policy (see Lafleur & Vintila, 2020; Saguin & Shivakoti, 2022) by showing how states of origin transnationally organize the implementation of social protection policies towards their emigrant population through CSOs and individual volunteers and thereby avoid interfering with destination countries’ sovereignty.

In the following section, I sketch out the framework of mediated citizenship as an approach to studying citizenship in practice and scrutinize how the socioeconomic marginalization of groups in India and their fragmented access to citizenship in their country of origin is intertwined with their precarious experience as labor migrants in the GCC countries, leading to the gaps between state institutions and marginalized groups which intermediators bridge with their representational practices. Next, I outline my research design, including case selection, respondent recruitment, the data collection process and analysis, and its limitations. In the subsequent empirical sections, I first give an overview of the CSOs’ structures and tasks before examining the three dimensions of mediated citizenship, namely representation, self-positioning and advocacy, and their implications as an informal representational practice.

## Conceptualizing state-citizen/immigrant relationships and institutional gaps in India and the GCC countries

The protection of citizens within a nation’s territorial borders is closely tied to the ideas of sovereignty and citizenship. Ensuring the safety and human rights of citizens living abroad creates a form of deterritorialized, transnational citizenship where the polity transcends national boundaries (Guillaume, 2019). Accessing social rights abroad, mainly as transnational social protection policies, for instance, through consular support, health and disability insurance schemes, is an essential dimension of transnational social citizenship, particularly in authoritarian countries that provide migrants with limited rights (Ruhs, 2013). Similar to how state-citizen relations extend beyond the borders of the nation-state through transnational citizenship policies, I conceptualize practices, such as the implementation of transnational social protection policies, as also taking place transnationally.

To study transnational citizenship practices, this article builds on substantive citizenship approaches, which overcome limitations of legalistic understandings by foregrounding the study of the day-to-day, lived experience of citizenship. Holston and Appudarai (1999, 4) state, “although in theory, full access to rights depends on membership, in practice that which constitutes citizenship substantively is often independent of its formal status.” Substantive approaches emphasize the constitutive role of informality in the form of personal networks that shape and structure citizen-state interactions, often due to the limitations of formal political systems (Berenschot and van Klinken, 2018). Central to the realization of rights are intermediaries and a variety of informal networks, which Piper and von Lieres (2015) refer to as ‘mediated citizenship’. In their framework, mediation is an attempt by a third party to overcome or take advantage of a representational gap between poor and marginalized groups and the state (Piper & von Lieres, 2015). Intermediaries’ objectives include one or several dimensions: 1) representation: representing a group to political authorities; 2)self-positioning: securing their role and interests; 3) advocacy: developing the political agency of a group, e.g., by building capacities to use their own voices (ibid.).

Piper and von Lieres’ (2015) framework echoes the role of intermediary actors as occupying the middle space between migrants and states (Deshingkar, 2019) described in the literature on migration brokerage (e.g., Gammeltoft-Hansen & Nyberg Sorensen, 2013; Lindquist et al., 2012). In this body of literature, brokers, which subsume a variety of formal and informal actors, facilitate migration and contribute to rescue and control functions of a transnational migration industry, spanning origin, transit, and destination countries (Gammeltoft-Hansen & Nyberg Sorensen, 2013). Whereas the brokerage scholarship is concerned foremostly with economic and cultural brokerage, the mediated citizenship framework also pays attention to political acts in brokerage, such as advocacy, awareness raising, and lobbying, and focuses on mediation practices as part of state-citizen relations (Piper & von Lieres, 2015), where economic gains and other forms of capital accumulation can, but do not have to motivate mediation. By adapting the concept of mediating citizenship, this article broadens the scope of previous literature, which predominantly used concepts of brokerage to explain the involvement of intermediary actors.

The relationship between intermediaries and the groups they claim to represent can take democratic, coercive, and clientelist articulations (Piper & von Lieres, 2015). Intermediaries are often migrants themselves, and as they are part of several networks (Lindquist et al., 2012), their modes of operation can vary situationally. As Hernández-León (2013, 34) states, “it is not uncommon for co-ethnics (…) who serve in immigrant advocacy organizations to subsequently establish legal advice, remittance, communications, and real estate firms catering specifically to immigrants”, thus pursuing sometimes contradictory objectives, such as advocacy and commercial interests. Similarly, as I will demonstrate later, distinctions between informal/formal roles and state and market (Lindquist et al., 2012) can be blurred as intermediaries may be state-employed or business owners relying on migrant labor.

Intermediaries’ third-party positions can be demarcated by their middle-class or elite social status; their reframing of issues to appeal to a broader (international) audience, and/ or the pursuit of interests not necessarily aligning with the social group’s (Piper & von Lieres, 2015). They claim to hold representative rights based, among others, on shared identities and interests, proximity to the issues of the represented group, or previous effective service provision. Mediated representation is an informal process not endorsed by legal frameworks; therefore, the legitimacy to speak and act on behalf of marginalized groups remains contestable (Piper & von Lieres, 2015).

In India, mediation is central to the everyday operations of the state (Berenschot, 2010). CSOs have taken up a significant position in “compensating for the state’s shortcomings in delivering the benefits of development” (Chandhoke, 2012, p. 42) to the extent that they developed as “an alternative to the state” (ibid.). Intermediaries can be individuals or CSOs who are often socio-economically better positioned than those they claim to represent (Piper & von Lieres, 2015). Notably, in India, activism in civil society is a defining marker of middle-class status (Harriss, 2006).

Drawing on Deshingkar (2019), I use the concept of precarity to describe the fragmented access to social, political, and economic rights and the ‘institutional gaps’ that Indian migrants in low-wage jobs in GCC countries experience. In South-South temporary contract migration, precarity affects the entire migratory cycle, impacting migrants both in origin and destination countries. It encompasses the root causes of migration, such as poverty and vulnerability, exploitative recruitment and employment practices during the migratory journey and the limited opportunities for economic and social reintegration upon return (Piper and Wither 2018; Deshingkar, 2019).

The majority of Indian migrants in the GCC countries fall under the so-called Emigration Check Required (ECR) category, which the Indian state assigns to migrants with a high school qualification below class 10 and implies working in low-wage employment in the GCC countries. Given the effects of social inequality on educational qualifications (see Mosse, 2018), it can be assumed that migrants in low-wage employment experienced socioeconomic marginalization in India (see also Wright, 2015). The religious and caste composition data of GCC-bound migrants from India is limited but indicates an increase in persons from marginalized groups (Kumar & Rajan, 2014), and in states such as Kerala and Telangana, a higher proportion of Muslim and Scheduled Caste^2^ migrants (see Kodoth, 2010; National Workers Welfare Trust, 2016). With stagnant formal employment growth in India and significant international wage differences, migration becomes a viable option for economic betterment (Ruhs, 2013; Piper & Withers, 2018). However, recruitment infrastructures are a major source of exploitation for emigrants, contributing to their vulnerabilities (Kathiravelu, 2016). Furthermore, the historically socioeconomic marginalization of groups that correspond with those who migrate into low-wage employment (see Jayal, 2009) and the constitutive role of informality in shaping and structuring citizen-state interactions (Berenschot, 2010) further limits Indian low-wage migrants to directly access state provisions in their country of origin. Historical forms of social inequality thus are reinforced by inequalities arising out of the transnational sphere of production and economic activity (see Jayal, 2009).

In the GCC region, the marginalization of Indian low-wage migrants is perpetuated by illiberal migration governance policies and practices, such as the kafala system^3^ – a temporary sponsorship system that is regarded as fostering exploitative labor conditions and offers no pathways to permanent residency or citizenship (at least for low-wage migrant workers) (Thiollet, 2019; Fernandez, 2021). Migrants in these countries lack fundamental political and social rights, including political association and free access to education and health services (Fernandez, 2021; Thiollet, 2019). The ability of low-wage migrant workers to legally address abuses is further constrained by inadequate official support, high litigation costs, and language barriers (Illias 2020).

Although the majority of immigrants are tied to temporary work visas in the GCC countries (Fernandez, 2021; Vora, 2013), migrants’ ability to navigate their migrant status differs dramatically, depending on their time of migration (Lori, 2022), social and economic capital, such as their nationality, social networks, and income (see Akıncı, 2022; Fernandez, 2021; Thiollet, 2019). White-collar workers from South Asian and Arab countries rank in the middle tiers, and persons in low-wage employment are at the bottom of the hierarchy (Fernandez, 2021). Moreover, not only do origin and destination state structures create marginalization, but non-national migrant elites play a significant role in the governance and exploitation of South Asian labor migrants (Vora, 2013). For example, in partnership with domestic economic elites, wealthy immigrants form business partnerships and subcontracting companies, often in the construction sector, where they employ other immigrants (Observation, Bahrain March 2022; Lori, 2022). This can serve as a form of boundary-making strategy through which immigrants, as employers, signal their social status by differentiating themselves from ‘migrant workers’ (Lori, 2022).

### Methods and research approach

This article investigates the reasons for, modes, and implications of mediation processes for welfare services and labor rights between migrant-run CSOs, individual volunteers, low-wage labor migrants, and state institutions. It thereby offers an empirical contribution to analyses of CSOs in the GCC region, which has received relatively less scholarly attention, also given the implications of an authoritarian context on researching civil society groups. For this study’s purpose, the sampling of CSOs focused on transnationally organized, Indian migrant-run organizations, which work on social welfare, labor protections, monitoring, and legal rights (see Malit & Al Youha, 2014). Indian migrants also represent the most significant proportion of the total migrant population stock in the GCC countries overall and in each country (KNOMAD, 2018). As I explained in the previous sections, I decided to study Indian migrant-run organizations as a case study because the character of state-citizen relations is particularly apt for studying mediating practices. I also chose to include one organization where Bahraini nationals and migrants of different nationalities participate, as it is one of the major groups working on behalf of migrants’ issues, and a majority of the volunteers and the migrants they support are Indians.

The difference in how CSOs have evolved creates in-case variations. At times, the CSOs are government-run, when the origin countries’ embassies initiate a support group, as in the case of the Indian Community Relief Fund in Bahrain. They can also be transnational extensions of existing informal networks in the country of origin, such as religious, caste, or town networks, or formed within the destination context around federal state/language identities, such as the Kerala Association, or gender categories, e.g., the Indian Ladies’ Association. The migrant-support organizations in this study also vary with regard to their organizational and/or financial links to states and official recognition. They can be broadly divided into those registered with the respective embassies or the destination states and unofficial/unlicensed CSOs. I talked to organizations that raise funds through fundraisers, membership fees, and private donations, including by royal family members, thus limiting impartiality to states and other major donors, who are often Indian businesspeople. Usually, the organizations’ members elect the governing boards, except for the Indian Community Relief Fund in Bahrain, where the Indian embassy selects the executive committee.

The article draws on multi-sited qualitative research between March 2020 and April 2022, including interviews, newspaper articles, brochures published by the organizations under study, and observations of meetings and interventions of migrant support CSOs on behalf of low-wage migrant workers. In recruiting respondents in the GCC countries, I initially used lists of migrant support organizations published on the Indian embassies’ websites; these are officially registered organizations. I aimed to extend the sampling frame to include organizations that were not necessarily listed as a form of “anti-snowball sampling’’ (Ackerly & True, 2008, p. 697) to include positions that might be excluded because of their opposition to the current administration. I did so by contacting Indian migrant organizations on social media, which are not always registered, and snowballing, using my network of previous respondents, colleagues, and acquaintances.

In pre-pandemic India, I held two in-person interviews with individuals who have been active in supporting low-wage migrant workers in the GCC countries and their repatriation to India; one of them previously had been residing in Oman. Organizations often rely on individuals and CSOs in the country of origin for administrative and bureaucratic purposes, as well as to coordinate logistical and financial issues with migrants’ families. I conducted eight semi-structured online interviews with representatives of migrant support organizations located in Bahrain, Dubai, Qatar, and Kuwait in February and March 2022. A three-week-long stay in Dubai and Bahrain in the spring of 2022 allowed me to participate in interventions that migrant support groups organized, such as medical camps, voucher distributions, and a mediated confrontation between a recruitment agency and its clients. In addition, I visited the migrant CSOs’ offices and met representatives in their day jobs’ localities, which helped me to situate the organizations and their representatives. In Dubai and Bahrain, I conducted 14 in-person, unrecorded interviews, of which I took extensive notes. Besides these interviews, I had several informal conversations, e.g., during shared car rides and on social media, which gave me insights into the respondents’ backgrounds and motivations. Furthermore, I talked informally with migrants in low-wage employment whom I met during the organized interventions, as well as with taxi drivers, construction workers, and employees in the hospitality sector whom I encountered on a range of topics, including their migration trajectory, working conditions, and aspirations.

All respondents were middle- or upper-middle-class, apparent by their occupations and other signifiers, such as family reunification, which requires a minimum income (see Fernandez, 2021). Two respondents work in high-rank executive positions for the Bahraini state. While most respondents hold Indian nationality, several are Bahraini, Kuwaiti, or Sri Lankan nationals. In a few cases, they acquired citizenship of their country of residence through naturalization, which is possible only for a minimal fraction of migrants, primarily for elites (see Vora, 2013). In their mid-twenties to late seventies, the respondents have lived in their respective destination countries between 15 and 55 years. Among those who retired, several returned to India but are still active in their organizations’ networks. About half of my respondents identify as female, but overall, based on my observations, Indian migrant-run organizations in the GCC context are male-dominated. Many of the respondents I talked to are community leaders beyond the immediate field of migrant-support organizations; they hold leadership positions in religious communities or head professional associations. Respondents often joined migrant-run CSOs through these networks. The work in the migrant-support groups is voluntary, meaning they are not paid for their undertakings.

The authoritarian context of the research influenced and limited the data collection process in several ways: the responsiveness and visibility of organizations that are not registered affected the sampling of organizations. They can be particularly subjected to state policing (see Mali and Al Youha 2014) and, therefore, might have been reluctant to engage with me. For my respondents’ and my safety, I did not record any of the in-person interviews I conducted in the GCC region. Similar considerations influenced my decision on where to do fieldwork: Bahrain’s civil society has been one of the most developed among the GCC countries (Kinninmont & Sirri, 2014), which reflects on the presence and visibility of migrant-led organizations.

My positionality as a White, Western European, younger woman mediated my interactions with respondents in several ways and often helped me gain access, e.g., by quickly building a good rapport with the female respondents and meeting them in private environments. Furthermore, possessing a European passport and working at a renowned European university facilitated in-person research as I could secure the needed visas and had the necessary financial means. Building trust was essential to secure appointments and being invited to meetings: the fact that I had conducted previous research and could show familiarity with relevant stakeholders in India also proved helpful. My impression was that respondents saw that my working knowledge of Hindi and Bengali expressed genuine interest and thus enhanced my credibility. It helped in understanding communication between volunteers and migrant workers, which is mainly in Hindi, which is neither the mother language of most volunteers nor migrants but is used as a *lingua franca* among South Asian migrants in the Gulf region.

For the data analysis, I applied an abductive approach, which combines inductive and deductive reasoning (Schwartz-Shea & Yanow, 2012). As I am interested in mediation practices, my interview guide questions and observations aimed to capture participants’ interactions and positionings vis-à-vis migrant workers and state institutions, and their understanding and choices of their practices. Among other questions, I asked about their tasks as volunteers, their interactions with state authorities, why they started volunteering, and their explanations for why there was a need for their inventions. I initially coded using categories based on the interview guides’ questions and my observations and then built categories inductively during the coding process as well deductively from other relevant literature I read.

## Mediating on behalf of migrants in low-wage employment: modes of operation

This section discusses the several aspects of mediation: representation, self-positioning, and advocacy, and how they play out empirically in the context of this research. The first subsection provides a brief overview of how the organizations under study are structured and the kind of tasks volunteers perform. Next, the discussion illustrates the explanations given by volunteers for the responsibilities that embassy representatives delegate to them. The following subsection discusses the implications of representation through CSOs: possible faster resolutions and access to institutions for migrants. The next two subsections engage with the dimension of self-positioning in the volunteers’ mediating efforts, namely how mediation is underpinned by power imbalances between volunteers and the migrants they support and how it relates to building social capital. The final subsection addresses the limited advocacy work and its reasons.

## Organizations’ structures and tasks of volunteers

All organizations and individuals under study focus on social and humanitarian initiatives for temporary labor migrants, mainly from India. CSOs and individuals deal with governmental authorities in the destination context, such as labor regulation and immigration institutions and the migrant’s respective embassies or administrations in countries of origin. These interventions are often organized transnationally, where volunteers coordinate activities with volunteer counterparts in the country of origin, e.g., for repatriation cases. Some groups have representation in each of the Gulf countries and India, where they collectively organize online events for migrant workers.

Volunteers take up issues concerning financial support for medical treatment, return to the country of origin in case of unemployment or illness, and identify and reparate mortal remains. They mediate disputes between employer/recruitment agencies and migrant workers; organize medical camps and distribute food and hygiene items; provide legal advice and awareness seminars; psychological and medical advice. Individuals in each organization might have different tasks; more prominent organizations often are divided into sub-committees or groups. Often, individual volunteers are involved in multiple organizations, e.g., regional-based ones and those representing the country. In some cases, these groups collaborate, mainly concerning individual migrant ‘cases’.

## The work that the embassy does not want to do…

CSOs often work with consulates and embassies in the GCC countries or on their behalf. Volunteers of these CSOs take up roles usually embodied by embassy officials as the legal representatives of their nationals – correspondent to the representational dimension of mediated citizenship. The Indian Community Welfare Committee in Dubai, for example, is dealing with the release of Indian prisoners, “The ICWC [Indian Community Welfare Committee] members, as well as the Indian Consulate, have cultivated an excellent rapport with the jail authorities, who have (…) provided the necessary assistance,” and “[a]t times, it is the prison authorities who are informing us of cases which are pending.” (Menon, 2002).

Similar to organizations such as the Indian Community Welfare Committee in Dubai, Indian embassies also select individual volunteers as credible authorities to work on their behalf in lieu of embassy officials. For example, in Bahrain, respondents accompanied migrant workers to police stations where they are well-known and recognized by the police staff:

Within the Indian embassy community outreach program, I had an Indian community ID card. If I took an Indian worker, at least with that card, I got a lot more access. The embassy gave this card only to very few of us. (…) to people they recognized at the grassroots and front line, ready to do the work. And whom they had observed over a number of years of doing it – “We are constantly comforting Indian workers in front of the Bahraini government department at your request’’. In my case, the embassy would very often call me and tell me “will you take her to immigration directorate or the police station, or will you take her to the Ministry of Labor to lodge a labor complaint?’’. I said I had no problem doing it, but increasingly, the government started asking, “If you are coming from the embassy, show us your ID.’’ (Interview 1, Online, India/Bahrain, March 2022).

Similarly, members of CSOs in other GCC countries also act as intermediaries on behalf of the Indian embassy, thus holding a representational role, as the following excerpt shows. It describes the procedure of a rescue mission when female migrant workers are found without identity documents:

Sometimes they take the women into custody. Sometimes [the police] send them with us. Because sending women into custody is also a pain for the police. They cannot keep a woman inside the lock-up. They have to transfer her to the shelter or the prison. If she has to be transferred to a shelter or a prison, the police have to do a lot of paperwork. And they are least bothered by doing paperwork for an Asian woman. They will say, “Okay, an embassy official is there. You are the embassy. Take her.” (…). (Interview 9, Trivandrum, March, 2020)

Representing another example of how mediation unfolds, a respondent re-tells how they helped to bring domestic workers to the Indian embassy in one of the GCC countries:

There was an issue with a woman domestic worker. (…) The embassies cannot directly intervene in these issues; they have to go through the foreign office, then to the police, and the police have to take up the issue. But there is another way also. If I come across a case, I can inform the embassy, and the embassy trusts me. They can authorize me for a day or two to rescue this woman. (…) [I did the rescue] as a private person. It [the rescue not being approved by the foreign office being and carried out by the police but a private person] is unofficial and illegal, but I am ready to take that risk, and the embassy is ready to take that risk. So then, I used to get involved with rescue operations. (Interview 9, Trivandrum, March 2020)

The shared narrative regarding why volunteers perform the tasks mentioned above is that due to the high numbers of Indian nationals, Indian embassies could not keep up with all the issues. However, respondents also point out that the embassies of other origin countries with comparatively smaller migrant populations similarly involve volunteers. This implies that embassies do not face constraints merely due to high migrant populations but because of a lack of resources and inherently of will. For example, a respondent from Sri Lanka revealed that the Sri Lankan embassy in Bahrain would always approach them whenever a case was difficult to solve – legally, bureaucratically, or emotionally.^4^

Involving volunteers means that origin states can avoid conflict with the state authorities in the destination country by possibly intervening in their sovereignty, e.g., when picking up an absconding domestic worker from their employer’s house or in state officials’ interests. The distinction between public and private spheres is blurred in how states and the migration industry operate in the Gulf (Thiollet, 2019): state officials and ruling family members are often business owners, employing immigrants (see Thiollet, 2019). Similarly, embassies might want to avoid confrontation with wealthy immigrants who employ immigrants and are well-connected with economic and political elites in the destination country, as this following statement demonstrates:

I have dealt with several cases where the owner [employer] is some billionaire guy from Kerala with good connections with the government and excellent connections with the Indian embassy. The workers themselves tell me that “we go to the embassy, and they do not do anything because the owner has such power.” (Interview 5, Online, Bahrain, March 2022).

A CSO supporting migrants in Bahrain ran a shelter for women who left their employers or husbands but had to shut it down in 2019. Even though expressing regret about it, a long-term volunteer stated:

I am not totally unhappy that the shelter is shut down because we did find that it was becoming very convenient for the embassy to outsource the responsibility without payments. (…) Earlier it was just a phone call [to] us to pick up that girl and keep her there. That changed, and I am glad it changed, because, at the end of the day, when you send out your vulnerable workers to countries where the protection mechanisms are not exactly right and the enabling environment is very weak, you need to have better protection. (Interview 1, Online, India/Bahrain, March 2022)

## Mediation as beneficial for all?

Besides avoiding bureaucratic structures, the advantages for embassies to work together with individual volunteers and CSOs are their fluency in Indian regional languages and their close-knit, transnational networks and knowledge of what happens ‘on the ground’, taking on what they call a social worker’s role. Indian migrants’ networks exist around regional, religious, and party affiliations. As political organizations are not allowed in the GCC countries, they run as cultural organizations. Embassy staff are often not familiar with regional languages, but only with Hindi and English, and are not aware of undocumented migrants as the latter are not registered with the embassies:

When we say social workers, it is not legal in any country. We have certain cultural organizations in Arab countries. They operate a kind of charity wing. They are spread all over most Arab countries. Especially the South Indians (…) are networked well. Somebody, in one case I know, wrote a number; and wrote in Malayalam, saying that “I need help; please contact me.” She threw it outside the window to an ally. (…) some Keralan guy saw this paper. He took it (…) and then called her, “What happened?”. She said, “I am locked up here in this room. I am a migrant worker from Kerala. I want to go back”. The guy used to read the newspaper, so he called me and said that “There is a case like this [name], so would you please take it up”. Then I called her, and then I talked to the embassy. Like me, there are many other people. There are at least 25 to 45 people like me in [country name]. Many people are in Qatar, Kuwait, everywhere. And we all speak Hindi, Tamil, and Malayalam. We can understand a little bit Kannada and Telugu also. So, we have the attitude of going to the workers and help. And we will always be also well connected to the embassy. So that culture has helped us a lot to widen our network and reach out to our workers. (…). (Interview 9, Trivandrum, March 2020)

The precedent excerpt also shows that in some instances, migrants could not access the embassies without volunteers’ support, often due to their confinement in homes or the lack of public transportation to reach the embassies. In other cases, approaching embassies via volunteers as mediators enables low-wage migrant workers to gain quicker access and faster responsiveness to their concerns, given the mediators’ comparatively more significant influence on embassy officials:

Once the embassy registers the complaint, then it may take time. (…). He [the embassy official] cannot resolve all the complaints. He can just receive the complaints and register and tell the worker, “Chalo, I will sort it out. I will talk to the employer tomorrow. I will call, and we will get a solution.” (…) After two, three days, the worker will call the embassy and asks, “Is there any solution? Did you talk?” “We will do it; we will do it”. After two, three times that the promises are not kept, the workers will start calling me or somebody who can approach the embassy who has influence in the embassy. Then (… ) I will talk to the official. “Please, Sir, please do it. It is a request from my side.” When somebody like me calls, they [the embassy officials] are little bit frantic. Then it is, “Okay, okay, we will do it. We will call them”. (Interview 9, Trivandrum, March 2020)

Migrants also instrumentalize the leverage of organizations and their access to resources and institutions in other ways: volunteers of two different organizations were telling me about the case of Paravati^5^, a domestic worker, which they both had recently taken up. Individual volunteers described the case as time-intensive and complicated and were taking similar action but did not know about each other’s involvement and only learned through me about it.^6^ Some volunteers recognize low-wage migrant workers’ agency, e.g., by acknowledging their role as their families’ breadwinners, regarding them as well networked with other migrants in low-wage employment and helping each other out^7^, and also by seeing that low-wage migrant workers are often underestimated.^8^ They know that their social class influences their perceptions and establish commonality by identifying themselves as workers and recognizing that everyone came to the GCC region to better their lives (ibid.).

### Selected support and deciding deservingness

Drawing on intermediaries seems beneficial for all stakeholders involved. Instead of seeing informality as deviant and detrimental to citizenship, Berenschot and van Klinken (2018, 107) propose incorporating informality into citizenship accounts as it “constitutes an important form of political agency as it enables citizens to deal with its unresponsive and unpredictable state institutions”. However, third-party mediation can be (mis)used by mediators to pursue material interests, e.g., in favor of political support (Piper & von Lieres, 2015), or to re-(confirm) hierarchical orders through the display of power. The following sub-sections showcase the dynamics of self-positioning at work in the process of mediation.

The intermediaries’ influence to push for action underlines the access to services and, more generally, the disaggregation of Indian citizenship along class lines. Status is associated with knowing how to navigate access and whom to address with claims:

[T]he blue-collar workers approach the second secretary or the labor officer in the embassy. Others directly call for the first secretary or to the ambassador “I am so and so, CEO of this company; I have an issue with this company. He is not allowing me to go back. He is not settling my salary”. But the blue-collar workers do not know anything. They need my help or people like me who are there on the ground. (Interview 9, Trivandrum, March 2020)

Mediation also creates and reinforces social hierarchies among volunteers and the persons they claim to help. For example, in awareness classes and medical camps, volunteers lecture low-wage labor migrants about personal hygiene, proper nutrition, and saving money^9^ – towards what they perceive as a good citizen’s conduct. The perceived need to teach about hygiene and savings indicates that the migrant workers attending these classes unreasonably spend money and are unclean and deficient. Notably, “hygiene practices are intimately enmeshed in negotiations and boundary drawing in class/caste relations” (Jack et al., 2022, p. 818) as notions of clean/unclean or pure/polluted are closely associated with caste, where the practices of ‘untouchability’ derives from maintaining social boundaries and caste hierarchies.

Migrant organizations’ volunteer practices mirror the Indian state’s discourse that India’s representation and reputation occur through individual migrants (see Natarajan, 2022). As Natarajan (2022) writes, the postcolonial Indian state has viewed ‘unskilled’ migration – equalized with ‘low’ class and caste – as an embarrassment in the international realm as the polity regards it as the continuation of indentured migration. Consequently, it has tried to restrict the emigration of ‘lower’ caste and class migrants, first through a discretionary passport policy until 1967 (Natarajan, 2022) and from 1983 onwards by introducing a registration system (the so-called Emigration Check Required category). As the presence of Indian workers in low-wage employment in the GCC region cannot be made invisible (despite the often geographically separated accommodation sites), let alone stopped, migrant workers’ bodies are regulated and disciplined through practices of self-betterment. These forms of volunteering attempting to transform the ‘other’ while reconfirming hierarchies are also reminiscent of civilizing missions – “the attempt made by a (…) social group (…) to transform a subordinate society in its own self-image.” (Barth and Hobson 2020, 1). In this way, volunteering through educational and awareness classes can be best understood as manifestations of continuing patterns of social inequality and social hierarchies along the lines of caste and class.

CSOs supporting migrants and individuals within these organizations also possess discretionary power over the organizations’ budget and decide which migrant receives financial and other kinds of support. As several volunteers told me, ‘vetting’ or checking if cases are genuine is a crucial step in helping a person: “There are different categories of workers. Some are genuinely illiterate, and some are literate, and sort of know the system also. You have to be able to distinguish if somebody really got trapped or (…) trafficked or somebody who kept hoping that somebody would come and help. It was always a very gray area to us.”^10^ Consequently, volunteers and organizations can establish their own criteria for who is deserving and who is not. For example, in the context of missing migrant cases forwarded to partner organizations in the GCC countries by an Indian CSO, the selection is motivated by the migrant families’ party affiliation.^11^

To some extent, the selectivity of support is also inherently gendered: most Indian-run organizations are male-dominated, and implicitly, the volunteer actions focus on male workers, who are also more visible and accessible to reach because they often live in ‘worker camps’, which are demarcated areas, whereas female workers (often domestic workers), live in private homes and therefore are less visible. Organizations have established ‘Ladies’ wings’, where female volunteers cater specifically to female workers. On the other hand, shelters exclude male workers, and volunteers who sometimes host female ‘run-away’ workers at their private homes would not take on a male person. The Indian state views women as more vulnerable than men and accordingly has put a travel ban on low-wage women workers below thirty. Volunteers partly support this approach, while others see it critically for encouraging migration via forged passports or without a valid visa.^12^

## Do good and talk about it

It is well-established that migrants rely on existing networks to migrate to the GCC countries and find work, and in the process, they are prone to be exploited by recruitment agencies or other contacts facilitating the migration process. Indians in Dubai and Bahrain dominate specific economic sectors, such as textile, electronic, and jewelry shops, which are run as family firms, supermarkets, and private education, where they prefer to employ co-ethnics often in exploitative conditions (Vora, 2013). According to Vora (2013), through governing other migrant populations, Indian elites are complicit with the state in producing social and economic hierarchies benefiting both nationals and elite expatriates while maintaining a structure of labor migration significantly disadvantaging the majority of foreign residents.

I encountered several instances where volunteering in migrant support groups and gaining social, economic, and cultural capital from occupying a mediating position were entangled in ambiguous ways. In my encounters, the juxtaposition was perhaps most evident with an Indian medical specialist who volunteered in several migrant-led organizations, such as a regional group and the Indian consulate-general medical committee, and has successfully helped repatriate bedridden patients and the mortal remains of deceased to India. He simultaneously partners in a law firm specializing in insurance cases for migrants who have met with accidents. By answering my question about how he and his partner found cases, he revealed that through his volunteer work (as well as his day job at the hospital), he came across cases of migrant workers who neither themselves nor their families know how to claim their accident insurance; and who he thus referred to his law firm. His knowledge of migrant workers’ precariousness, combined with his insights into the insurance system, creates a system where the respondent can use his social capital for exploitable purposes.

When establishing contact with potential respondents, I found a lot of ‘success stories’ of low-wage migrant workers displayed on support organizations’ social media sites. Later, respondents continued sharing newspaper articles and posts with me via social media groups I was part of. Reporting on these stories functions to gain legitimacy for their work and, most importantly, to portray themselves as a ‘good-doer’ and successful, thus as a kind of self-positioning within the Indian community. However, when asked about their motivation to do this kind of volunteer work, a commonality among the respondents was that they were not volunteering to gain social prestige. However, some volunteers claim that others’ motivation precisely lies in acquiring social capital, as the following statements show:

Many volunteers are not doing this work for the right reasons. Some of them enjoy this, trying to show that they are these migrant heroes and are doing it for their self-publicity. I have come across people who just want to put it on Instagram, Facebook, or WhatsApp groups. Just give an initial rescue picture and do not bother about the worker. (Interview 1, Online, Bahrain/India February 2022)

[I]t is PR. To look good in the community (Interview 5, Online, Bahrain, March 2022).

It is common for volunteers to be members of multiple organizations. However, these organizations have similar agendas and do similar work, therefore raising the question of why volunteers must be part of multiple associations, especially if they are already highly involved with one organization, which means being available day and night and investing all of their free time to volunteer, sometimes to the extent of not seeing their families regularly.^13^ Volunteering for low-wage migrant workers is also a means to create and maintain visibility and opportunities for self-positioning and securing a role as a representative vis-à-vis state institutions and international organizations, thereby generating social prestige and capital. In addition, being part of several organizations allows for taking up a leadership position in one of them if the access to influence is limited in another organization. For example, leadership roles in the ICRF are limited because the Indian embassy elects the committee/leadership. For that reason, one of the respondents joined a newly created group to become chairman of the country wing. Later, he became a spokesperson on behalf of the Indian community to a UN organization through this position.

## Advocacy or charity work?

Advocacy work is one dimension of mediated citizenship, which Piper and von Lieres define as to “develop some form of active citizenship’’ (2015:704) and “political agency” (ibid) of marginalized groups. While Indian CSOs located in India and the GCC region, who are often transnationally organized, have been advocating for enhancing migrants’ rights (see Burmeister-Rudolph, 2024), these practices are often still representational, meaning intermediators speak and lobby on behalf of low-wage migrants. Although there are incidents where CSOs support migrants’ agency, e.g., by supporting their family members in India to run in regional elections to highlight the political negligence of migrants’ issues (see Burmeister-Rudolph, 2024), at least in the cases presented here, advocacy was limited to representational advocacy, such as being part of transnational migrant rights’ networks, such as Migrant Forum in Asia.

The space for criticizing and contesting the origin and destination states differs between volunteer groups. In some instances, such as for the organizations associated with the Indian embassies, which implement most of the Indian state’s transnational social protection schemes of the Indian state, the embassy selects the organizations’ leading committee and thereby can influence its mandate, even though the organizations raise funds. Although some of the volunteer groups claim that they have pushed for legislative change in the destination country where they are located, these groups simultaneously receive financial support from members of the ruling family, which casts doubt on their ability to contest the status quo. Consequently, rather than being a medium for societal transformation, volunteer organizations act as partners of origin and destination states and become accountable to them rather than to the people they claim to represent.

For the reasons mentioned above, from the perspective of some respondents, volunteering can turn into “charity work,”^14^ which does not change the structural conditions of migrants’ precarity but instead means “cleaning up after issues created by the companies and the governments”,^15^ leaving volunteers demoralized and disillusioned. In response, they turn to other articulations of engagement, such as creating awareness about migrant workers’ abuse through digital means, where they report individual stories and online campaigns pointing to structural issues. This form of activism is anonymous and limited to digital platforms. The sustainability of the volunteer work this article describes is also debatable because of a lack of a new generation of volunteers: many are retired or about to retire. Although it would be possible for most to continue living in the GCC countries after retirement, some of the respondents had already returned to their country of origin or were in the process. New(er) volunteer cannot or are not willing to be constantly available^16^ or do not want to risk losing their residential permit by confronting state authorities, such as the police.^17^ Support infrastructure, such as a shelter for female migrants, was closed for these reasons.^18^ As a result, migrants in precarious employment will be left standing alone, even more so than before, if the origin country embassies continue to outsource interventions and service provisions to volunteer groups while labor law reforms in the GCC countries remain not enforced (Fernandez, 2021) and, instead, de facto have a contrary effect of enhancing states’ migration control and limit migrants’ autonomy (Thiollet, 2019)

## Conclusion

CSOs and individual volunteers are key stakeholders in governing migration in the GCC countries, particularly in providing access to migrant-origin states’ transnational social protection policies and dealing with the destination countries’ labor regulation and law enforcement institutions. This article aimed to conceptually grasp and empirically scrutinize the role of these non-state stakeholders and the power dynamics in the interactions between state institutions, civil society actors, and migrants in low-wage employment. In the previous pages, drawing on the concept of mediated citizenship (Pieper and von Lieres 2015), the analysis highlighted the inner workings underlying the complex of volunteers operating through CSOs and individually as intermediaries. Building on substantive approaches to citizenship, I argued that a person’s fragmented access to social, political, and economic citizenship rights *in the country of origin*, who then will potentially emigrate into low-wage employment in the GCC countries, partially shapes the ‘necessity’ of volunteers’ mediation efforts. I demonstrated how complicated and sometimes impossible access to social policy schemes opens opportunities for persons with more significant resources, such as social capital, education, and leisure time, to become intermediaries and, in some instances, profile themselves as diaspora heroes. However, mediation not only bridges the gap caused by class and caste-based social inequalities but can reinforce social hierarchies between volunteers and low-wage employed migrants, who often share the same nationality. At the same time, it is a cost-effective strategy for origin state bureaucracies and a means to avoid confrontation with destination countries’ authorities. This study underlines that intermediaries’ objectives are entangled and messy, and particularly in an authoritarian environment, the various modes of mediation, such as representing, self-positioning, and advocating, are not easily distinguishable. By bringing together the concept of mediated citizenship and insights from the literature on migration brokerage, this article demonstrated that mediation takes place in a transnationally organized space, where migrants as intermediaries have to navigate origin and destination countries’ bureaucracies. Conceptually, it extends the framework of mediated citizenship by highlighting this transnational dimension; for scholarship on migration brokerage, this research shows how social rights are an (unlikely) object of mediating practices.

The analysis provided insights into the organizational forms and processes of migrant-supporting CSOs. A key observation of this research has been that, apart from a few exceptions, migrant-run CSOs’ support forms explicitly or implicitly around national identities. A volunteer who has been active in a multi-national support group states that care revolves around notions of a shared (national) identity. “[E]ven within [name of organization] you have people who are more (.) caring about their citizens. Maybe on the surface, it looks international but (…) you tend to see [name of nationality] mostly dealing with [name of country] cases. Everyone speaks English (…) I think it is more kinship stuff than language.’’^19^

Beyond Indian organizations, which were the focus of this article, migrant-led CSOs from other origin countries undertake similar forms of informal representation. However, involvement levels differ between national communities: respondents stated that, e.g., the Philippines’ embassy officials relied less on volunteers and solved matters directly with the destination state’s authorities. On the other hand, despite Bangladeshis making up a large workforce in the GCC countries, Bangladeshi volunteers’ presence is limited^20^ or perhaps less visible. Future research could further develop this article’s insight to comparatively explore why the migrant organizations’ presence and mediation practices differ and why they predominantly organize around national identities in the first place.

## Data Availability

More detailed information on the data is available from the author upon reasonable request. For data protection reasons, this excludes full interviews and transcriptions.

## Abbreviations

GCC: Gulf Cooperation Council
CSOs: Civil society organizations

## Footnotes

1 I define CSOs as “all non-market and nonstate organizations outside of the family in which people organize themselves to pursue shared interests in the public domain” (Tomlinson, 2013).

2 Scheduled Caste is an official designation by the Indian state, subsuming a list of castes considered to have been historically marginalized because of practices of untouchability (Mosse, 2018).

3 In Bahrain, the kafala system was dismantled in 2009. Instead of private sponsors, the Labor Market Regulatory Authority has been the sponsor of all immigrants (Thiollet, 2019). In all GCC countries domestic workers, who are large part of migrants in low-wage employment, are exempted from labor law regulations (Thiollet, 2019).

4 Unrecorded, in-person follow-up conversation with respondent, Bahrain, April 2022.

5 Name is changed.

6 Unrecorded Interview, Bahrain, March 2022; Unrecorded Interview, Bahrain, April 2022.

7 Unrecorded Interview Bahrain, April 2022.

8 Unrecorded Interview, Bahrain, March 2022.

9 Observation, Bahrain March 2022.

10 Interview 1, Online, Bahrain/India, March 2022.

11 Observations, Trivandrum, March 2020.

12 Interview 11, Hyderabad, March 2020; Unrecorded interviews, Bahrain, March 2022.

13 Several unrecorded conversations, Bahrain, March/April 2022; Interview 9, Online, Kuwait, March 2022.

14 Interview 5, Online, Bahrain, March 2022.

15 Interview 5, Online, Bahrain, March 2022.

16 Interview 5, Online, Bahrain, March 2022.

17 Interview 1, Online, Bahrain/India, February 2022.

18 Interview 1, Online, Bahrain/India, February 2022.

19 Interview ID05, Online, Bahrain, March 2022.

20 Interview ID05, Online, Bahrain, March 2022.
